# Association of Neuroimaging Markers on Clinical CT Scans With Domain-Specific Cognitive Impairment in the Early and Later Poststroke Stages

**DOI:** 10.1212/WNL.0000000000207756

**Published:** 2023-10-24

**Authors:** Georgina Hobden, Margaret J. Moore, Emma Colbourne, Sarah T. Pendlebury, Nele Demeyere

**Affiliations:** From the Department of Experimental Psychology (G.H., N.D.), University of Oxford, United Kingdom; Queensland Brain Institute (M.J.M.), University of Queensland, Australia; Nuffield Department of Clinical Neurosciences (E.C., S.T.P., N.D.), University of Oxford; and NIHR Oxford Biomedical Research Centre and Departments of General (Internal) Medicine and Geratology (S.T.P.), John Radcliffe Hospital, Oxford, United Kingdom.

## Abstract

**Background and Objectives:**

Poststroke cognitive impairment (PSCI) is associated with neuroimaging markers, including cortical atrophy and white matter lesions (WMLs), on clinically acquired CT neuroimaging. The objective was to investigate the association between cortical atrophy/WMLs and PSCI in specific cognitive domains in the acute/subacute and chronic stages after stroke, to provide clarity on the relationship between these neuroimaging markers and the temporal evolution of PSCI.

**Methods:**

We visually assessed cortical atrophy using the Global Cortical Atrophy (GCA) scale and WMLs using the Fazekas scale. Oxford Cognitive Screen or Birmingham Cognitive Screen assessed PSCI at 2 time points (acute/subacute and chronic) in 6 domains (language, memory, number processing, executive function, attention, and praxis). We binarized domain-specific performance as impaired/unimpaired using normative cutoffs. Multivariable linear and logistic regression analyses evaluated associations between GCA/Fazekas scores with acute/subacute and chronic global and domain-specific PSCI, and ANCOVAs examined whether these scores were significantly different in patients with recovered vs persistent PSCI. Age, sex, education, NIHSS, lesion volume, and recurrent stroke were covariates in these analyses.

**Results:**

Among 411 stroke patients (Mdn/IQR age = 76.16/66.84–83.47; 193 female; 346 ischemic stroke; 107 recurrent stroke), GCA and Fazekas scores were not associated with global cognitive impairment in the acute/subacute stage after stroke, but GCA score was associated with chronic global PSCI (*B* = 0.01, *p* < 0.001, 95% CI 0.00–0.01). In domain-specific analyses, GCA score was associated with chronic impairment in the memory (*B* = 0.06, *p* < 0.001, 95% CI 0.03–0.10) and attention (*B* = 0.05, *p* = 0.003, 95% CI 0.02–0.09) domains, and in patients with persistent PSCI, these domains showed significantly higher GCA scores than patients who had recovered (memory: *F*(1, 157) = 6.63, *p* = 0.01, *η*^*2*^*G* = 0.04; attention: *F*(1, 268) = 10.66, *p* = 0.001, *η*^*2*^*G =* 0.04).

**Discussion:**

This study highlights the potential effect of cortical atrophy on the cognitive recovery process after stroke and demonstrates the prognostic utility of CT neuroimaging for poststroke cognitive outcomes. Clinical neuroimaging could help identify patients at long-term risk of PSCI during acute hospitalization.


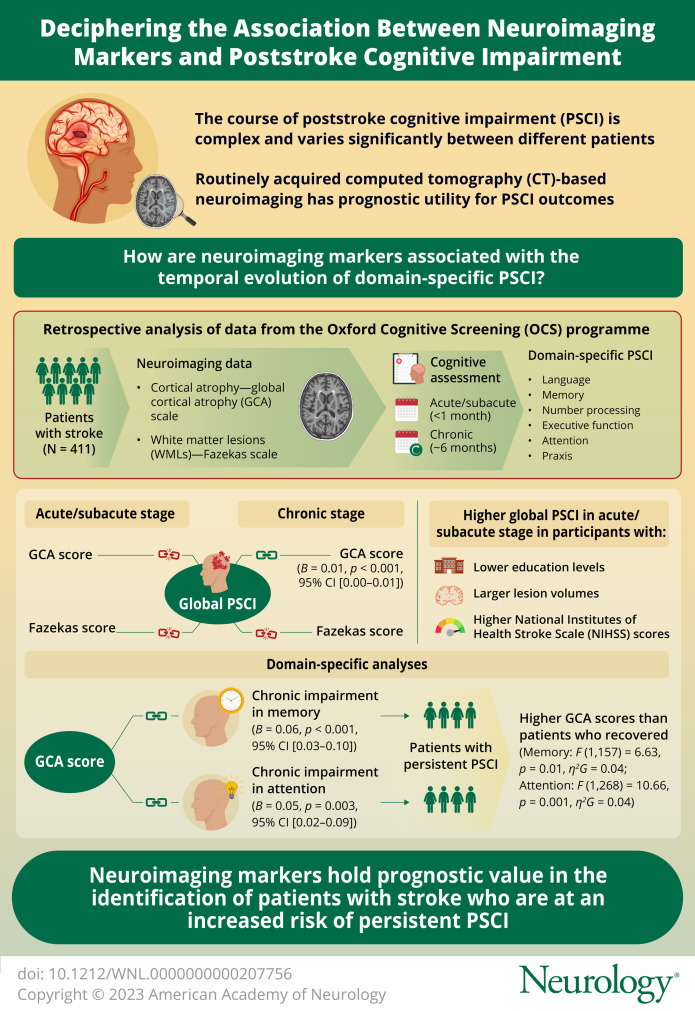


## Introduction

Stroke is a leading cause of disability worldwide^1,2^ and poststroke cognitive impairment (PSCI) is highly prevalent, with an estimated 38% of stroke survivors experiencing PSCI in the first year after stroke.^3^ PSCI is associated with reduced quality of life^4,5^ and an increased risk of depression,^6,7^ highlighting the importance of identifying patients at risk of PSCI during acute hospitalization, as earlier identification of at-risk patients may improve future care planning and discussions around prognosis.

Clinical guidelines require diagnostic brain imaging to be performed for each suspected stroke patient.^8-10^ Although MRI produces higher-resolution scans, CT presents practical benefits over MRI (e.g., faster, lower cost, and fewer contraindications) and, therefore, remains the standard imaging modality in clinical settings.^11-13^ Therefore, investigating whether neuroimaging markers on CT are associated with PSCI is of substantial clinical and academic importance, as CT imaging represents a routinely acquired source of data with potential prognostic utility for PSCI.^12^

Previous research supports the possibility that routinely acquired CT imaging has prognostic utility for PSCI. White matter lesions (WMLs) on CT are associated with a 3-fold increase in the likelihood of developing PSCI, and the presence of cortical atrophy and WMLs is associated with a 2-fold to 3-fold increase in the risk of developing poststroke dementia.^12^ However, there remain substantial unanswered questions around these associations, particularly for the relationship between neuroimaging markers and the temporal evolution of domain-specific PSCI.

The temporal evolution of PSCI is complex and heterogeneous across patients. Although poststroke cognitive impairment is highly prevalent in the acute stage after stroke, longer-term recovery trajectories are variable, with some patients remaining cognitively impaired in the chronic stage after stroke and others recovering.^14^ Determining whether neuroimaging markers on CT imaging are associated with persistent cognitive impairment after stroke may have important clinical implications, by enabling identification of ‘at-risk’ patients using routinely collected data. Nevertheless, most previous studies have assessed PSCI at only one time point.^12^

Previous studies have also tended to use brief cognitive screening tools (e.g., t-MMSE)^15^ that classify patients as “impaired” or “not impaired” at a broad level of cognitive functioning, without delineating between different domain-specific cognitive impairments (e.g., visuospatial neglect and apraxia).^12,16-18^ In clinical practice, cognitive screening tools designed specifically for stroke populations (e.g., Oxford Cognitive Screen [OCS]^19^) are increasingly recommended,^20,21^ as they offer advantages over brief screening. Domain-specific screening is more sensitive to different types of domain-specific PSCI^22^ and facilitates more nuanced understanding of cognitive status in different domains, which offers advantages for tailoring care packages and supporting return to work/education. Routinely acquired domain-specific cognitive screening data may offer valuable insight into the relationship between neuroimaging markers and domain-specific PSCI.

To clarify the association between neuroimaging markers and the temporal evolution of domain-specific PSCI, this study investigated the relationship between cortical atrophy and WMLs on CT with global and domain-specific PSCI at 2 time points poststroke (acute/subacute and chronic). Both neuroimaging and acute/subacute cognitive data were acquired as part of routine clinical care for stroke, enabling investigation of cognitive functioning in multiple different domains within the same large clinical sample.

## Methods

### Standard Protocol Approvals, Registrations, and Patient Consents

This study retrospectively analyzed data collected from the OCS program. This program recruited a sample of stroke survivors during acute hospitalization. Stroke survivors were then followed up at home approximately 6 months after discharge. The OCS program received ethical approval from the NHS (OCS-Tablet and OCS-Recovery studies: NHS RECs 14/LO/0648 and 18/SC/0550). All participants in the OCS program provided informed consent (written or witnessed) when they were recruited and then at follow-up.

### Participants

The OCS program recruited stroke survivors from the acute stroke unit at Oxford University Hospital through convenience sampling. Inclusion criteria for the OCS program were clinical diagnosis of stroke, ability to provide written/witnessed informed consent to participate in research, ability to remain alert for at least 20 minutes (as judged by multidisciplinary team in the stroke unit), sufficient level of English required for the OCS, aged at least 18 years. Exclusion criteria were visual and/or hearing impairment limiting the ability to complete cognitive tasks. Participants were recruited to the OCS program during 2 recruitment periods: wave 1 (March 2012 to January 2015) and wave 2 (February 2015 to March 2020). Participants were visited at home after discharge to complete a follow-up cognitive assessment. This was completed approximately 6 months after their recruitment date.

Stroke survivors originally recruited to the OCS program were included in this study if they met the following additional inclusion and exclusion criteria. Inclusion criteria were follow-up cognitive assessment at 6 months poststroke with the OCS or Birmingham Cognitive Screen (BCoS)^23^ (n = 430) and a usable CT head scan from within 14 days poststroke (n = 411). Exclusion criteria were CT scans exhibiting significant movement artefacts, partial brain coverage, or poor resolution, which precluded assessment of WMLs and/or cortical atrophy (n = 19). Table 1 provides demographic and clinical details of the sample obtained from medical records.

**Table 1:**
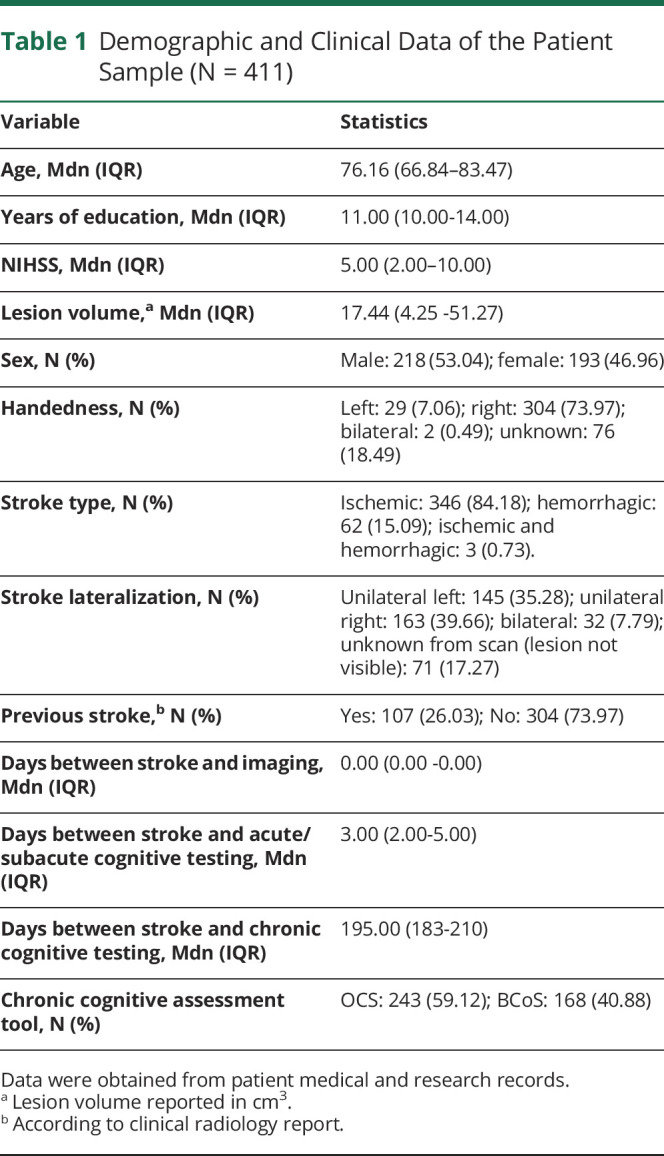
Demographic and Clinical Data of the Patient Sample (N = 411)

Variable	Statistics
Age, Mdn (IQR)	76.16 (66.84–83.47)
Years of education, Mdn (IQR)	11.00 (10.00-14.00)
NIHSS, Mdn (IQR)	5.00 (2.00–10.00)
Lesion volume,^a^ Mdn (IQR)	17.44 (4.25 -51.27)
Sex, N (%)	Male: 218 (53.04); female: 193 (46.96)
Handedness, N (%)	Left: 29 (7.06); right: 304 (73.97); bilateral: 2 (0.49); unknown: 76 (18.49)
Stroke type, N (%)	Ischemic: 346 (84.18); hemorrhagic: 62 (15.09); ischemic and hemorrhagic: 3 (0.73).
Stroke lateralization, N (%)	Unilateral left: 145 (35.28); unilateral right: 163 (39.66); bilateral: 32 (7.79); unknown from scan (lesion not visible): 71 (17.27)
Previous stroke,^b^ N (%)	Yes: 107 (26.03); No: 304 (73.97)
Days between stroke and imaging, Mdn (IQR)	0.00 (0.00 -0.00)
Days between stroke and acute/subacute cognitive testing, Mdn (IQR)	3.00 (2.00-5.00)
Days between stroke and chronic cognitive testing, Mdn (IQR)	195.00 (183-210)
Chronic cognitive assessment tool, N (%)	OCS: 243 (59.12); BCoS: 168 (40.88)

Data were obtained from patient medical and research records.

a Lesion volume reported in cm^3^.

b According to clinical radiology report.

### Demographic and Clinical Data

Data on age, sex, years of education, NIHSS, and recurrent stroke were collected during acute hospital admission by clinical staff. Data on participant ethnicity and first language were not collected. These data were collected using an electronic patient record system.

### Cognitive Data

PSCI was assessed for all participants during both the acute/subacute (<1 month poststroke) and chronic (∼6 months poststroke) stages using stroke-specific cognitive screening tools designed to be inclusive for stroke patients with visual impairments, motor impairments, neglect, and aphasia. Occupational therapists and clinical researchers assessed acute/subacute PSCI for all patients during hospitalization using the OCS. Clinical researchers only assessed chronic PSCI during home follow-up visits using either the OCS *(*n = 243) or the BCoS (n = 169). All assessors completed formal training, and all assessments are standardized, ensuring consistency and replicability of cognitive results. All participants tested during the first recruitment wave (March 2012 to January 2015) used the BCoS at follow-up. The OCS was used at follow-up for all subsequently recruited participants (February 2015 to March 2020) because of a change in study protocol. Notably, the BCoS takes substantially longer time to administer than the OCS. However, this length difference is largely due to the BCoS including several tasks that are not included in the OCS (e.g., figure copying, auditory working memory). These BCoS-only tasks were not included in this analysis. Instead, only tasks that have direct analogs in both the BCoS and OCS (e.g., picture naming, cancellation) were included in this study. Previous research suggests that these analogous OCS/BCoS tasks tap the same underlying cognitive functions and do not significantly differ in sensitivity.^24,25^

Performance in each subtask of the OCS/BCoS was binarized as impaired/not impaired because of the small range of values available for most tasks (e.g., range of scores for picture naming = 0–4) and because OCS/BCoS tasks are designed to screen for the presence vs absence of PSCI, rather than quantifying PSCI severity. We classified performance in each task as impaired if the participant scored below the established cutoff score for the task, which was determined using normative data from neurologically healthy adults.^19^ From these impairment classifications, we determined global and domain-specific PSCI for each participant in the acute/subacute and chronic stages poststroke. We calculated global PSCI as the number of tasks on which the participant was impaired, divided by the number of tasks completed. For domain-specific cognitive impairment, we classified participants as impaired in a cognitive domain if they were impaired in at least one subtask designed to assess that domain at the relevant time point (eTable 1, links.lww.com/WNL/D88).

Finally, for patients who showed domain-specific PSCI in the acute/subacute stage poststroke, we assessed their domain-specific cognitive recovery. Patients were classified as having recovered from a domain-specific cognitive impairment if they were domain-impaired in the acute/subacute stage poststroke, and not domain-impaired in the chronic stage poststroke. Patients with the same domain-specific cognitive impairment in the acute/subacute and chronic stages poststroke were classified as having a persistent cognitive impairment in that domain.

### Imaging Data

Whole-brain noncontrast CT scans (slice thickness 5 mm) were acquired as part of routine clinical care during acute hospitalization. Participants provided consent for scans to be accessed and used for research purposes. An in-house radiographer (Oxford University Hospital) and members of the research team (M.J.M. and G.H.) conducted quality control of CT images to ensure their suitability for inclusion in this study.

GH visually assessed cortical atrophy and WMLs on axial CT slices for all participants. GH completed visual ratings while blind to demographic and clinical information. Visual assessments of cortical atrophy used the Global Cortical Atrophy (GCA) scale,^26^ which is reliable for CT.^27^ The GCA scale evaluates cortical atrophy in 13 brain regions overall (eTable 2, links.lww.com/WNL/D88) by giving each region an atrophy score of 0 (absent), 1 (mild), 2 (moderate), or 3 (severe). If it was not possible to score a region because of a visible stroke lesion, this region was given the same score as the region in the opposite hemisphere.^28^ Total GCA score was calculated by summing the scores from all regions (range = 0–39).

Visual assessments of WMLs used the Fazekas scale,^29^ which has moderate reliability on CT.^30^ The Fazekas scale evaluates WMLs in deep white matter and periventricular white matter. Periventricular WMLs were rated as 0 (absent), 1 (symmetrical caps), 2 (smooth halo), or 3 (irregular hypoattenuations extending into deep white matter), and deep WMHs are rated as 0 (absent), 1 (punctuate foci), 2 (beginning confluence of foci), or 3 (large confluent areas).^29^ Total Fazekas score was calculated by summing the scores from both regions (range = 0–6).

We assessed intrarater and interrater reliability of the cortical atrophy and WML ratings using a randomly sampled subset of the imaging data set (n = 100). GH rated the imaging subset twice at an interval of 4 months to confirm intrarater reliability while blind to original visual rating scores. EC also rated the imaging subset to confirm interrater reliability. EC was blind to visual ratings by GH and vice versa.

To control for stroke lesion volume in statistical analyses, the MRIcron software package (McCausland Center for Brain Imaging, Columbia, SC) was used to manually delineate lesions on native space CT scans using a standardized processing procedure.^31^ However, this was only possible where the lesion was visible on the CT scans. Although all participants had a clinical diagnosis of stroke, the use of acute CT imaging for this study meant that the infarction was not always visible or defined sufficiently for manual delineation. G. Hobden and M.J. Moore completed the manual delineations while blind to demographic and clinical data. The lesion masks were smoothed (5 mm full width at half maximum in the z-direction) and then binarized (thresholded at 0.5). The Statistical Parametric Mapping 12 and Clinical Toolbox were used to reorient scans and lesion masks to the anterior commissure and warp them into 1 × 1 × 1 mm stereotaxic space. M.J. Moore visually inspected the normalized scans and lesion masks for quality. Lesion masks that passed this quality control procedure were used to calculate stroke lesion volume.

### Statistical Analysis

Statistical analyses were conducted using R-Studio (4.0.2). We conducted basic descriptive analyses to investigate the cognitive and neuroimaging data. We assessed normality of variables using the Shapiro-Wilk test. Given the ordinal nature of rating scale data, Spearman rank correlation coefficient analyzed intrarater and interrater reliability of the total GCA score and total Fazekas score for the randomly selected imaging subset.^32^ Missing data were imputed using the MICE package.^33^

Next, multivariable linear regression analyses investigated the relationship between neuroimaging markers on CT (cortical atrophy and WMLs) and global PSCI in the acute/subacute and chronic stages poststroke. The outcome variable was the proportion of cognitive tasks impaired, and the predictor variables were total GCA score and total Fazekas score.

Next, multivariable logistic regression analyses investigated domain-specific PSCI in the acute/subacute and chronic stages poststroke. Separate regression analyses were conducted for each cognitive domain at each time point. Domain-specific impairment (impaired vs not impaired) was the outcome variable, and total cortical atrophy/total WMLs were predictor variables. Multivariable linear and logistic regression analyses included all participants in the sample (N = 411).

**Figure 1:**
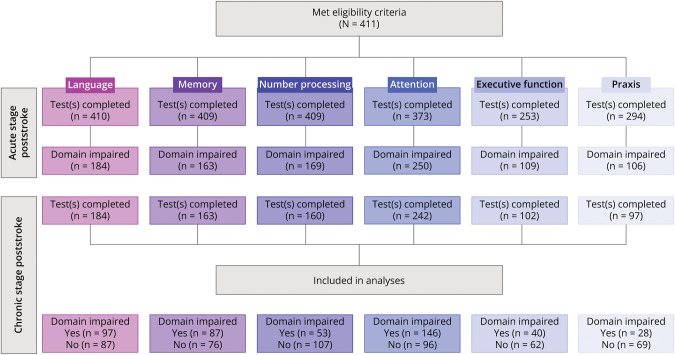
Patient Inclusion and Cognitive Impairment Incidences by Domain for the Present Study The first row shows the number of patients who completed the relevant OCS task(s) to assess each cognitive domain in the acute/subacute stage poststroke. The second row shows the number of patients classified as impaired in each cognitive domain in the acute/subacute stage poststroke. Third and fourth rows show the number of patients who completed cognitive assessment at the chronic stage and impairment incidences by domain.

Next, one-way between-subjects ANCOVAs investigated whether cortical atrophy and WMLs were associated with domain-specific cognitive recovery. A separate ANCOVA was conducted for each cognitive domain that showed associations with total GCA score and/or total Fazekas score in the previous regression analyses. Each ANCOVA compared total GCA/Fazekas scores for participants who had recovered from acute/subacute cognitive impairment in the chronic stage poststroke (i.e., impaired acute/subacutely, not impaired chronically), vs participants who had not recovered (i.e., impaired both acute/subacutely and chronically). These analyses included only participants from the original sample who were classified as domain-impaired in the acute/subacute stage poststroke and who had completed the subtasks designed to assess that cognitive domain in the chronic stage poststroke. Figure 1 presents a flowchart showing how inclusion for these analyses was determined.

All regression analyses and ANCOVAs included years of age, sex, years of education, NIHSS, stroke lesion volume, and recurrent stroke as covariates. Days between stroke and chronic cognitive assessment was a covariate in analyses of chronic cognitive impairment. Covariates were selected based on review of previous literature pertaining to predictors of poststroke cognitive impairment.^34,35^ We corrected for multiple comparisons using the Bonferroni correction. We did not conduct a priori power analyses as we used all available cognitive and neuroimaging data. Alternative analyses that excluded patients with missing data, including lesion volume and NIHSS data, are presented in Supplementary Materials (links.lww.com/WNL/D88).

### Data Availability

Anonymized demographic, clinical, and cognitive data not published within this article will be made available by request from qualified investigators. Ethical restrictions prevent the sharing of brain scans.

## Results

This study included a total of 411 stroke survivors, including 193 (46.96%) female stroke survivors. Participants were aged on average 76.16 years (IQR = 66.84–83.47) and had an average of 11 years of education (IQR = 10–14). A total of 304 (73.97%) participants were right-handed, and 275 (66.90%) stroke survivors had a visible ischemic lesion on CT brain imaging, 62 (15.09%) had a visible hemorrhage, 3 (0.73%) had both a visible acute ischemic lesion and hemorrhage, and 71 (17.27%) had no visible lesion but had a confirmed clinical diagnosis of ischemic stroke. There were 145 (35.28%) unilateral left lesions and 163 (39.66%) unilateral right lesions. There were 107 (26.03%) participants who had a previous stroke lesion visible on CT imaging (i.e., recurrent stroke).

The median time between stroke and acute/subacute cognitive assessment was 3 days (IQR = 2.00–5.00). The median time between stroke and chronic cognitive assessment was 195 days (IQR = 183–210). The median number of cognitive tasks impaired for participants was 2 (IQR = 1–5) in the acute/subacute stage after stroke and 1 (IQR = 0–3) in the chronic stage after stroke. Table 2 reports individual OCS task completion rates and impairment incidence rates for the acute/subacute and chronic stages poststroke. Figure 1 shows cognitive domain impairment incidence rates for the acute/subacute and chronic stages poststroke. eTable 1 (links.lww.com/WNL/D88) outlines the tasks used to assess each cognitive domain.

**Table 2:**
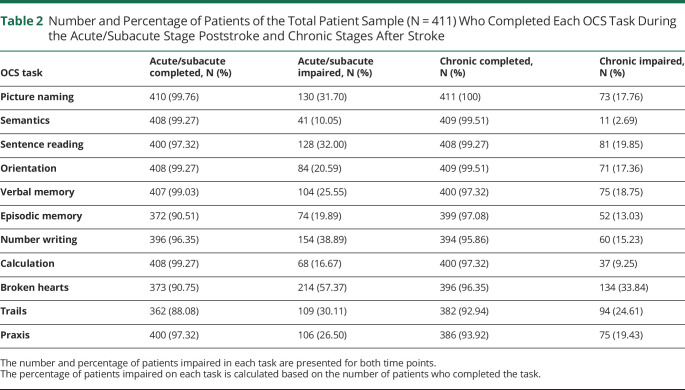
Number and Percentage of Patients of the Total Patient Sample (N = 411) Who Completed Each OCS Task During the Acute/Subacute Stage Poststroke and Chronic Stages After Stroke

OCS task	Acute/subacute completed, N (%)	Acute/subacute impaired, N (%)	Chronic completed, N (%)	Chronic impaired, N (%)
Picture naming	410 (99.76)	130 (31.70)	411 (100)	73 (17.76)
Semantics	408 (99.27)	41 (10.05)	409 (99.51)	11 (2.69)
Sentence reading	400 (97.32)	128 (32.00)	408 (99.27)	81 (19.85)
Orientation	408 (99.27)	84 (20.59)	409 (99.51)	71 (17.36)
Verbal memory	407 (99.03)	104 (25.55)	400 (97.32)	75 (18.75)
Episodic memory	372 (90.51)	74 (19.89)	399 (97.08)	52 (13.03)
Number writing	396 (96.35)	154 (38.89)	394 (95.86)	60 (15.23)
Calculation	408 (99.27)	68 (16.67)	400 (97.32)	37 (9.25)
Broken hearts	373 (90.75)	214 (57.37)	396 (96.35)	134 (33.84)
Trails	362 (88.08)	109 (30.11)	382 (92.94)	94 (24.61)
Praxis	400 (97.32)	106 (26.50)	386 (93.92)	75 (19.43)

The number and percentage of patients impaired in each task are presented for both time points.

The percentage of patients impaired on each task is calculated based on the number of patients who completed the task.

The median number of days between stroke and CT neuroimaging was 0 (IQR = 0–0). The median stroke lesion volume was 17.44 cm^3^ (IQR = 4.25–51.27). The median total GCA score was 15 (IQR = 8–21), and the median total Fazekas score was 2 (IQR = 0–3). The Spearman test showed a moderate positive correlation between total GCA scores and total Fazekas scores (rho = 0.56, *p* < 0.001).

The Spearman test showed a strong positive intrarater correlation between the total GCA scores (rho = 0.98, *p* < 0.001) and total Fazekas scores (rho = 0.90, *p* < 0.001) and a strong positive interrater correlation between total GCA scores (rho = 0.88, *p* < 0.001) and total Fazekas scores (rho = 0.77, *p* < 0.001). A detailed analysis of intrarater and interrater reliability on clinically acquired CT brain imaging is presented in the study by Hobden et al.^27^

### Acute/Subacute PSCI

Linear regression analysis showed that acute/subacute global PSCI was not associated with either total GCA score or total Fazekas score. However, participants with lower levels of education (*B* = −0.02, *p* < 0.001, 95% CI −0.02 to −0.00]), higher NIHSS scores (*B* = 0.01, *p* = 0.013, 95% CI 0.00–0.01), and larger lesion volumes (*B* = 0.00, *p* < 0.001, 95% CI 0.00–0.00) showed increased global PSCI in the acute/subacute stage poststroke.

Logistic regressions showed that acute/subacute domain-specific PSCI was not associated with total GCA scores or total Fazekas scores for any of the 6 cognitive domains assessed after correction for multiple comparisons (adjusted *α* = 0.008). However, acute/subacute language impairment was associated with larger lesion volumes (*B* = 0.01, *p* < 0.001, 95% CI 0.00–0.01) as was acute/subacute attention impairment (*B* = 0.01, *p* < 0.001, 95% CI 0.01–0.02), acute/subacute executive function impairment (*B* = 0.01, *p* < 0.001, 95% CI 0.00–0.01), and acute/subacute number processing impairment (*B* = 0.01, *p* = 0.007, 95% CI 0.00–0.01). Years of education was associated with language impairment (*B* = −0.11, *p* = 0.001, 95% CI −0.18 to −0.05), memory impairment (*B* = −0.09, *p* = 0.006, 95% CI −0.16 to −0.03), and number processing impairment (*B* = −0.11, *p* = 0.001, 95% CI −0.18 to −0.05). Other covariates were not significant.

### Chronic PSCI

**Figure 2:**
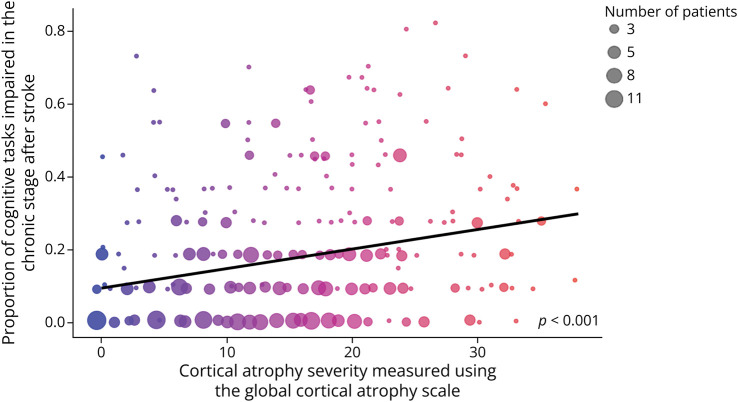
Association Between Cortical Atrophy (X-Axis) and Global Poststroke Cognitive Impairment in the Chronic Stage After Stroke (Y-Axis) Cortical atrophy was measured using the Global Cortical Atrophy (GCA) scale. Total GCA scores are presented along the x-axis (range: 0–39). Global cognitive impairment was operationalized as the number of cognitive tasks on which a patient was classified as impaired, relative to normative data, divided by the number of cognitive tasks completed in the chronic stage after stroke (i.e., proportion of tasks impaired). Given the large number of participants included in this study (n = 411), circle size is used to represent the number of patients that occupy each point on the plot. Point color provides an additional visualization of GCA score.

Linear regression analysis showed that global PSCI in the chronic stage after stroke was associated with total GCA score but not total Fazekas score. Participants with more severe cortical atrophy (i.e., higher total GCA score) showed a higher level of global PSCI than participants with less severe cortical atrophy (*B* = 0.01, *p* < 0.001, 95% CI 0.00–0.01). This association is presented in Figure 2. Participants with fewer years of education also showed increased global PSCI in the chronic stage poststroke (*B* = −0.02, *p* < 0.001, 95% CI −0.00 to −0.01). Other covariates were not significant predictors of chronic global PSCI.

Logistic regressions showed that higher total GCA scores were associated with chronic domain-specific PSCI in the memory (*B* = 0.06, *p* < 0.001, 95% CI 0.03–0.10) and attention (*B* = 0.05, *p* = 0.003, 95% CI 0.02–0.09) domains after correction for multiple comparisons (adjusted *α* = 0.008). Chronic domain-specific PSCI was not significantly associated with total Fazekas score in any of the cognitive domains assessed. Years of education was significantly associated with chronic PSCI in the language (*B* = −0.13, *p* = 0.001, 95% CI −0.21 to −0.05) and memory (*B* = −0.12, *p* = 0.002, 95% CI −0.20 to −0.5) domains. Other covariates were not significantly associated after multiple comparison correction.

### Cognitive Recovery

**Figure 3:**
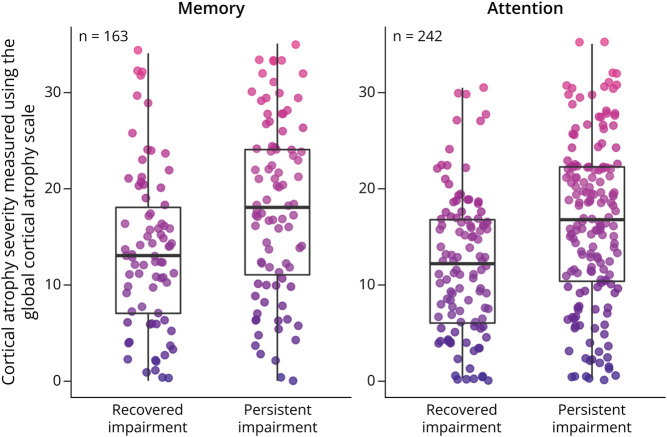
Association Between Cortical Atrophy and Cognitive Recovery in the Memory and Attention Domains Cortical atrophy severity (y-axis: total GCA score) are shown for patients with persistent domain-specific cognitive impairments (i.e., impaired in the acute/subacute and chronic stage after stroke) vs recovered cognitive impairments (i.e., impaired in the acute/subacute stage only) (x-axis). The number of patients (n) categorized as domain-impaired in the acute/subacute stage poststroke is shown for each cognitive domain. Values are presented for individual patients (points) and the means, interquartile ranges, and minimums/maximums for each subsample (boxplots). Point color provides an additional visualization of GCA score.

Given the observed associations between global cortical atrophy and chronic domain-specific PSCI in memory and attention, we ran ANCOVAs to compare total GCA scores in participants with recovered vs persistent impairments in these domains. A total of 163 and 242 patients were included in the memory and attention analyses, respectively. ANCOVAs showed that patients with persistent impairments (i.e., impaired at the acute/subacute stage and chronic stage) in the memory domain had significantly more severe cortical atrophy (i.e., higher total GCA scores) than patients who had recovered from acute/subacute memory impairments (i.e., not impaired at chronic stage) (*F*(1, 157) = 6.63, *p* = 0.01, *η*^*2*^*G* = 0.04). Patients with persistent impairments in the attention domain also had significantly more severe cortical atrophy than patients who had recovered from acute/subacute attention impairments (*F*(1, 268) = 10.66, *p* = 0.001, *η*^*2*^*G =* 0.04). These associations are presented in Figure 3.

## Discussion

This study investigated the association between cortical atrophy and WMLs on clinically acquired CT brain imaging and PSCI at 2 time points in a large sample of stroke survivors recruited from a regional stroke unit. Cortical atrophy and WMLs were not associated with either global or domain-specific PSCI in the acute/subacute stage after stroke. However, more severe cortical atrophy was associated with increased global PSCI in the chronic stage, as well as domain-specific impairments in memory and attention, independent of age, sex, education, NIHSS score, and lesion volume. Furthermore and crucially, memory and attention impairments were more likely to persist into the chronic stage poststroke in patients with more severe cortical atrophy, indicating a potential relationship between cortical atrophy and domain-specific cognitive recovery after stroke.

This study builds on established literature by demonstrating that cortical atrophy severity may be associated with cognitive recovery patterns after stroke by increasing the risk of persistent cognitive impairment in a potentially domain-specific manner. The results showed that patients with cognitive impairments specifically affecting memory and attention that persisted into the chronic stage after stroke had significantly more severe cortical atrophy than patients who had recovered from acute/subacute cognitive impairments in these domains. This is a novel finding that stems from our ability to analyze CT neuroimaging data alongside domain-specific cognitive data from multiple time points after stroke, in contrast to previous studies that have conducted brief cognitive screening at a single time point.^12,16,17^ Although the pathophysiologic mechanisms linking cortical atrophy to increased risk of persistent cognitive impairment are unclear from this study, stroke infarcts may interact with cortical atrophy to exacerbate poststroke cognitive decline or impede neural recovery mechanisms. Interactions between neurodegenerative and vascular imaging markers have similarly been proposed to explain poor cognitive outcomes in nonstroke populations, including patients with Alzheimer disease,^36^ symptomatic atherosclerotic disease,^37^ and cerebral small vessel disease^38^ and older adults.^39^ Nevertheless, additional research is needed to determine whether this is the case for stroke.

This study found no association between WMLs and PSCI in either the early or later stages after stroke, which was unexpected, given that previous CT studies have reported a link between increased WML severity and higher risk of PSCI, particularly in the executive function domain.^18,40-43^ Nevertheless, our null findings (i.e., absence of evidence) do not necessarily provide evidence of absence (i.e., no relationship between WMLs and PSCI). Instead, it is possible that the CT scans, visual rating measure, and/or the cognitive tasks lacked sensitivity compared with those used in previous research. For example, Hobden et al.^28^ observed a relationship between WML severity and executive functioning in a similar patient cohort but used both a more sensitive measure of WML severity (Age-related White Matter Changes scale^44^) and a more sensitive measure of executive function (OCS-Plus Rule Finding task^45^). Alternatively, these null results may reflect our conservative approach to multiple comparison correction or lack of statistical power stemming from the relatively low number of patients who completed executive function testing (Figure 1), relative to other cognitive domains. This lower completion rate may be due to the executive function tasks placing greater demands on motor skills, which are often impaired after stroke, compared with other cognitive tasks used in this study.

There are several limitations in this study. First, we aimed to include a clinically representative stroke patient sample from a clinical stroke unit, but there was likely some selection bias because of the requirement for follow-up assessment and informed consent. Second, visual ratings of cortical atrophy and WMLs on clinically acquired CT imaging may have been complicated by the presence of cerebral edema, which is common in the acute/subacute stages poststroke. In addition, electronic patient records were used to collect data on some variables (e.g., NIHSS, handedness), but data were not always digitally recorded and collecting NIHSS data was not a routine part of clinical care at the outset of data collection in 2012. This resulted in a high rate of data missingness as did the use of hyperacute CT imaging, which hampered our ability to calculate lesion volume in some cases because CT has low sensitivity to ischemic lesions at this stage after stroke.^46^ While we attempted to mitigate this limitation using a robust imputation method,^47^ imputation may have affected results of the analyses. Indeed, there were subtle differences between results of our analyses with imputation and those completed in subsamples of participants with complete data (see Supplementary Materials, links.lww.com/WNL/D88). In addition, this study may have lacked power to detect associations between poststroke cognitive impairment and important variables, such as recurrent vs first-ever stroke. Research using larger samples with more complete data is required to provide more conclusive evidence as to the predictive value of these covariates. Furthermore, this study attempted to control for stroke lesion effects by including lesion volume as a covariate. However, the relationship between other potentially related lesion characteristics on cognitive outcomes was not considered — for example, lesion lateralization or territory. Nevertheless, although this study focused heavily on neurodegenerative rather than lesion-based imaging markers, previous research offers important insight into the effect of lesion location on domain-specific cognitive outcomes after stroke.^48^ Finally, many other clinical, demographic, and socioeconomic factors can potentially help predict the occurrence and persistence of poststroke cognitive impairments.^34,35^ For example, past research has demonstrated that depression acts as a strong predictor of cognitive impairment in chronic stroke.^34,49^ As this study was not able to assess all potentially relevant predictors, future research can aim to investigate whether atrophy and WML data add prognostic value over and above other established outcome predictors.

Future research should attempt to incorporate clinically acquired neuroimaging markers (e.g., atrophy severity, stroke lesion characteristics) into a cognitive outcome prediction model, designed to provide clinicians and patients with an individualized estimate of the likelihood of recovering from acute/subacute PSCI. Identification of at-risk patients during acute hospitalization may influence follow-up care. For example, the presence of substantial cortical atrophy on clinically acquired CT imaging may trigger follow-up cognitive reviews after patient discharge from the acute clinical setting. More broadly, understanding patients' risk for cognitive decline is important for health care professionals when engaging in discussions about prognosis with patients and their families. Future research is required to investigate how health care professionals should engage patients and their families in these conversations and the type of information that should be provided.

Overall, the present investigation highlights the prognostic utility of clinically acquired CT neuroimaging by demonstrating that cortical atrophy on CT may be a risk factor for persistent poststroke cognitive impairment, specifically in memory and attention. This has important implications for clinical practice because identifying patients who are at an increased risk of persistent PSCI could influence follow-up cognitive care, particularly if these patients are identified during acute hospitalization.

## Appendix Authors

**Table:**

Name	Location	Contribution
Georgina Hobden, MSc	Department of Experimental Psychology, University of Oxford, United Kingdom	Conceived the framework for this study, analysed and interpreted the data, and prepared the manuscript for submission
Margaret J. Moore, DPhil	Queensland Brain Institute, University of Queensland, Australia	Collected data for the study, delineated the lesion masks and assisted with data analysis and manuscript preparation
Emma Colbourne, MEng	Nuffield Department of Clinical Neurosciences, University of Oxford, United Kingdom	Involved in brain scan rating
Sarah T. Pendlebury, DPhil	Nuffield Department of Clinical Neurosciences, University of Oxford; NIHR Oxford Biomedical Research Centre and Departments of General (Internal) Medicine and Geratology, John Radcliffe Hospital, United Kingdom	Critically reviewed and edited the manuscript
Nele Demeyere, PhD	Department of Experimental Psychology; Nuffield Department of Clinical Neurosciences, University of Oxford, United Kingdom	Supervised the study, acquired the funding, provided oversight and project administration and critically reviewed and edited the manuscript

## Glossary

BCoS: Birmingham Cognitive Screen
GCA: Global Cortical Atrophy
OCS: Oxford Cognitive Screen
PSCI: poststroke cognitive impairment
WMLs: white matter lesions
